# The importance of residual renal function in chronic dialysed patients

**Published:** 2009-04-25

**Authors:** D Rădulescu, D Ferechide

**Affiliations:** * ‘Carol Davila’University of Medicine and Pharmacy, Nephrology Clinic,‘Sf. Ioan’ Clinical Emergency Hospital, Bucharest Romania; ** Department of Physiology, ‘Carol Davila’ University of Medicine and Pharmacy, Bucharest Romania

## Abstract

In the last decade, many researches have reached to the conclusion that preservation of residual renal function (RRF) is important after initiating dialysis, as well as in the predialysis period.

RRF has been proven to contribue to the quality of life of dialysis patients. Longer preservation of RRF provides a better small and middle molecule removal, improved volemic status and arterial pressure control, diminished risk of vascular and valvular calcification due to better phosphate removal. Deterioration of RRF results in worsening of anemia, inflammation and malnutrition. It is now proven a direct relationship between RRF value and survival in dialysis patient.

Several therapeutical intervention have been proven to ameliorate the decline of RRF in dialysis patients. Some of them are identical with those before initiating dialysis: ACE–inhibitors and/or angiotensin–receptor blockers, limiting the use of nephrotoxic drugs, avoiding contrast media procedures, adequate control of blood pressure. Others are specific for dialysis period: adequate dialysis dose, avoiding excessive ultrafiltration, preventing arterial hypotension during dialysis sessions, using biocompatible dialysis membranes, ultrapure water for dialysis, dietary interventions.

## Measurement of RRF

The exact value of RRF is necessary both at the moment of initiating dialysis therapy and on the course of the dialytic therapy in order to adjust, simultaneous with the RRF decrease, the dose of dialysis.

The value of remaining diuresis do nor correlate with RRF[1.

The inuline clearance is the standard method to which other GFR measurements are compared, but it is expensive, especially when it has to be repetead at regular intervals during the dialytic therapy[2

The creatinine clearance is frequently used in current practice for GFR estimation, but it has limits: the creatinine depends not only on the glomerular filtration rate, but also on the muscular mass and individual's age; within the kidney, besides free glomerular filtration, creatinine suffers tubular secretion too, which becomes significant as the renal function deteriorates; in uremics, creatinine extrarenal (intestinal) elimination is present too. In addition, the usual method of measuring plasma creatinine (with alkaline picrat) may give results that are falsely higher because of the non–creatinine cromogens. As a result, creatinine clearance, calculated by the classical formula UxV/P, overestimates the GFR real value[1].

The blood urea or urea clearance are even less precise for GFR estimation. The production of urea depends on diet, proteic catabolism, and the excretion is influenced by diuresis. Within the kidney, urea, after glomerular filtration, suffers tubular reabsorption. Urea clearance underestimates the real value of GFR.

Cockcroft and Gault formula for creatinine clearance has been developed in order to exceed the limits of the classical formula (UxV/P),but this formula allows a prediction only for the endogene creatinine, not for the GFR[3, 4].The inhibiton of the creatinine tubular secretion with cimetidine may provide assessments close to the real ones, but the necessary doses are very high and there can't be achieved a complete blocking of tubular secretion. In addition,for hemodialysed patients, the blocking of tubular secretion of creatinine by cimetidine won't take effect [3]

MDRD formula (modification of diet in renal disease) is not usefull in estimating RRF in dialysed patients, because it overestimates the real values, sometimes with 100%[5].

Clearance of beta2 microglobulin or cistatine may represent an alternative of RRF estimation, because they are excreted only by glomerula filtration, but their use in dialysed patients hasn't come into the current use yet^6^,].

GFR isotopic markers –^125^I–iothalamate,^99m^Tc–DTPA or ^51^Cr–EDTA–have the advantage of simplicity and avoid urine collection, but may overestimate real GFR as they are eliminated by extrarenal routes too and have a large volume of distribution; besides, they are expensive[7].The overestimation of real GFR, by approximately 20% in patients with normal renal function,increases as the renal function deteriorates. Iohexol clearance represents a method of GFR value estimation without a risk of affecting the remaining kidney function, but it is also expensive[8]

Numerous studies show a good correlation between the arithmetic mean of creatinine and urea clearance and RRF real value[7];clearances are calculated by the classical formula (UxV/P). This method is cheap and easy to repeat, depending only on patient's compliance. For HD patients, plasma values for urea and creatinine are measured one of the days between the dialysis sessions, when urine is collected for 24 hours.

## RRF and mortality risk in dialysis patients

Several researches demonstrated that preservation of RRF is associated with better survival rate, both in PD and HD patients. 

CThe first research emphasizing that RRF has an influence on survival of PD patients was performed in 1995 by Maiorca[9], who studied RRF as an independent factor, demonstrating that the persistence of a significant renal clearance is accompanied by a longer survival in PD patients. Subsequent studies[10,11,12,]. revealed that RRF and not the dialysis dose is predictive for a longer survival of PD patients. CANUSA study (Canada–USA Peritoneal Dialysis Study Group), whose results were published in 1996[13]and which started with the premise of an equivalence between RRF and PD clearance, demonstrated that the sum of the two clearances (RRF + PD) for small molecules is a predictor irrespective of other factors for the mortality of PD patients. Reanalysis[14,]of the data of CANUSA study in 2001 showed that RRF and not the dose of dialysis is the one that directly influences patients' survival.

Other researches [15,16] demonstrated the same relation between RRF and survival rate in HD patients.

In 2002, the ADEMEX (ADEMEX = ADEquacy of Peritoneal Dialysis in MEXico) study, performed in PD patients, has reached to the conclusion that residual renal clearance and the dialytic clearance are not equivalent and additive [17].Increasing solvite clearance in DP was not accompanied with better survival rate in overall or anuric patients, demonstrating that RRF was the one that directly influenced patients' survival rate. This observation led to the conclusion that preserving RRF has additional metabolic benefits, beyond better low molecular solvites removal. Indeed, subsequent studies[18]demonstrated an increased frequency in anurics, compared to patients with preserved RRF, of numerous metabolic and cardiovascular complications: more severe anemia, increased frequency of erythropoietin resistance, higher CaxP product, increased rate of malnutrition, inflammation, and ventricular hypertrophy.

Impact of RRF upon volemic status and cardiac hypertrophy.

Extracellular liquid volume is increased in peritoneal dialyzed patients with residual GFR below 2mL/min than in patients with residual GFR above 2mL/min[19]. In the reanalyse of CANUSA study [14],every 250mL of urine was associated with 36% reduction in global mortality for PD patients. These data–indirectly–emphasized that the kidney, even in advanced stages of functional insufficiency, has a major importance in eliminating water and sodium.Subsequently, the study done by Ates and collaborators[20].confirmed that the value of sodium and water fractionalexcretion has a predictive value for the mortality of PD patients.

PD patients with history of hidrosaline retention show degrees of more severe hypertrophy and cardiac dilatation, as well as more important alternation both of systolic and diastolic function than in the patients with controlled volemia, as other recent studies demonstrate[21,22].Considering that cardiac hypertrophy is an important factor of mortality prediction for chronically dialysed population, the data above suggest that cardiovascular complications,which are more frequent among anuric dialysed patients, are, at least partly, due to the inefficient control of volemia after RRF loss. In PD patients, arterial hypertension  is more difficult to control as RRF decreases [22,23]

In addition, the same studies note that as RRF decreases, other complications appear; the anemia becomes more severe (with increased erythropoietin needs), hypoalbuminemia is aggravating, the arterial pressure pulse increases. All those data suggest that RRF influence on cardiac hypertrophy is due not only to water and salt elimination, but also to other effects such as a better purification of uremic toxins. In predialysis CRF patients, left ventricle (LV) mass increases parallel with the decrease in residual GFR [24]; initiating PD led to the regression of left ventricular hypertrophy (LVH) and improvement of the cardiac function [25]. The evidence that residual renal clearance for solvents with low MW and not the peritoneal clearance is the one that directly influences LVH [22], as well as the fact that LVH regresses post–transplant[26], permitted the observation that there are certain non–dialysing uremic toxins which mediates LVH in peritoneal dialysed patients.

There are a few studies [27,28] referring to RRF influence on volemia and cardiovascular status in HD patients. All demonstrate a directly proportional relation between RRF preservation and the control of volume–dependent hypertension and volemia.

## RRF and calcium–phosphate balance

Most of the studies demonstrate a better phosphate control in PD versus HD [29,30] as a result of better removal of phosphate and better preservation of RRF. In PD patients the presence of hyperphosphatemy is closely related with RRF rate: only 1/3 of the patients with preserved RRF show increased values of seric phosphates, while over 1/2 of anurics have hyperphosphatemy. Anurics also have an increased inflammatory status; the association of hyperphosphatemia and hypercalcemia leads to the increase of valvular calcification risk, vascular wall stiffening, high degree of cardiac hypertrophy [30]. 

A study published by Wang and collab. in 2005 in Nephrology Dialysis Transplantation [31] connects valvular calcification predisposition to fetuin–A depletion, a circulatory inhibitor of calcification and a negative reactant of the acute stage. On the other hand, the authors did not find an association between fetuin–A depletion and RRF reduction and they conclude that in anuric peritoneal dialysed patients, fetuin–A depletion is not responsible for increased frequency of valvular calcification.

## Inflammation and RRF

The presence of inflammation is noted with increased frequency (between 12–65%) in chronic dialysed patients [32]. The inflammation degree, estimated by C-reactive protein [33 ,34] or interleukin–6 dosing [35,36] directly influences dialysed patients' survival rate and cardiovascular mortality. In predialysis uremic patients it was reported an inversely proportional relation between RRF and plasma concentration of pro-inflammatory mediators [ 40]. Similar results were published in PD patients: RRF decrease is associated with the increase of inflammatory response [39] estimated by C–reactive protein dosing [37] or by the sanguin level of a soluble vascular cell adhesion molecule [38]. All the studies indicate that the relation between RRF and the degree of inflammation is independent of patient's cardiovascular status [41]. The mechanism through which RRF loss worsens the inflammation of chronically dialysed patients seems to be, as some studies on animals indicate, oxidative stress of vascular endothelium with the activation of monocytes and cytokines[42,43]. A vicious circle takes place: inflammation worsens,at its turn, the deterioration of RRF [44]. The association of inflammation with LVH and RRF loss has an additive effect on cardiovascular complications rate in dialysed patients [39].

## The contribution of RRF to the nutritional status

Malnutrition is frequent in chronic dialysed patients and represents an independent factor which influences mortality, especially by cardiovascular diseases. The preservation of diuresis and implicitly of a significant RRF permits a more liberal hygieno–dietetic regimen. Using systems of nutrition estimation that are subjective [45,46,47] – questionnaires on alimentary supply, good condition, etc or objective– dry body mass, seric albuminemia [47,48], most of the studies concluded that the proteic and energetic dietary amount, as well as the vitamins dietary amount are inversely correlated with RRF value. Other researches demonstrated a direct relation, independent of the dialysis dose, between RRF reduction and the appearance of malnutrition, which suggests that native kidney removes some non–dialysable uremic toxins with medium MW.

RRF loss is also accompanied by a increased resting energy expenditure [49], which can lead to malnutrition unless there is a compensatory increase in energetic and proteic dietary regimen. The general and cardiovascular mortality risk correlates with increased basal energetic expenditure. The determinant link in the relation malnutrition–inflammation–atherosclerosis–increased cardiovascular mortality seems to be the loss of RRF; increasing the dialytic clearance has no benefits.

## The importance of RRF in removal of uremic toxines

Preservation of a significant RRF allows a better removal of uremic toxins with medium molecular weight. Irrespective of the dialysis type, beta 2 microglobulin level is lower in dialysed patients with preserved RRF [50,51,52]. In anuric PD patients, increasing the dialysis dose is followed by a better removal of toxins with low MW, but not of those with medium MW and other toxins that circulate bound to proteins–such a s P–cresol [53,54].

## FRR and quality of life in dialysed patients

Considering all the factors that favorably influence RRF preservation in chronic dialysed patients, one may conclude that the quality of life, not only the survival period is ameliorated. The patient with preserved RRF has a more liberal diet, a better compliance to potassium and hidrosaline restrictions or to the drug regimens. A lower rate of complications needs less drugs, which has better psychological and financial impact. A better social and familial insertion is achieved, the sensation of handicap which is present in most dialysed patients due to the dependency upon extrarenal purification therapy is diminished or absent. Of course, there is a large individual variability which especially depends on patient's age and existing co–morbidities; the advanced age, the coexistence of generalized manifestations of atherosclerosis, predialitic cardiovascular diseases, etc are a few examples in which the RRF influence on chronic dialysed patient's life is insignificant.

NECOSAD study [55] demonstrated, in PD patients, a positive influence of RRF preservation on the most dimensions of life quality: physical functions, vitality, uremic symptoms, sleeping disorders; in the same study, the dialysis clearance had no influence on these dimensions.

##  Preservation of RRF in chronic dialysed patients

All the researches performed until present [56,57, 58,59] indicate a better preservation of RRF in PD versus HD,which gave birth to the concept of ‘integrative care approach’ of CRF patient: patients with preserved RRF will initially be oriented to PD and, after losing RRF, transferred on HD.

A retrospective study performed in 2000 on a large number of patients (‘Van BW, Vanholder RC, Veys Net al’. An evaluation of an integrative care approach for end–stage renal disease patients. ‘**J Am Soc Nephrol** 2000’)demonstrated that such an attitude is accompanied by a better survival rate in PD patients transferred on HD as compared to those that remained on PD or as compared to those who begun on HD from the beginning [60]. 

The superiority of PD in preserving RRF can be explained through two factors:

- Better hemodynamic stability in PD, which decreases renal ischemic aggressions. In 2000, Moist demonstrated that higher values of postdialytic medium hypertension are associated with a better RRF preservation in chronic HD [59]. In NECOSAD study [55], there was demonstrated a relation between the frequency of intradialytic hypotension episods and the rate of RRF decline; the periods of volemic depletion were associated with a more rapid deterioration of RRF.
- Nephrotoxic effects of the pro–inflammatory mediators released within the extracorporeal circuit of HD.

On the other hand, recent researches [61,62] demonstrated that, using biocompatible hemodialysis membranes and ultrapure water, RRF decline is similar to the one in the continuous ambulatory peritoneal dialysis. RRF decrease is more rapid in patients hemodialysed with cellulose membranes as compared to patients hemodialysed with high-flux polisulfone [63].

Foreign substances present within incompatible membranes, in contact with blood, stimulate mononuclear and complement activating [63].

Some recent studies [64,65] suggest a more rapid deterioration of RRF in patients receiving automatic peritoneal dialysis (APD) versus those on continuous ambulatory peritoneal dialysis (CAPD) and explain this through the intermittent nature of automatic peritoneal dialysis (APD) which produces a osmotic and volemic chargeless steady than in CAPD. Other researches [59,66, 67] consider these observations as groundless because of the non-uniform selection of patients in ADP.

Irrespective of the type of PD, the rate of RRF decline is correlated with the frequency of peritonitis episodes [44] and with the type of dialysis solution.

Avoiding nephrotoxic drugs–non–steroidal anti–inflammatories, aminoglicosides etc– is not only a predialytic measure of preventing CRI progression, but it also must be done after the dialysis initiation in order to preserve RRF. If investigations with contrast media are needed, all the prophylactic measures must be taken [68]: adequate hydration (eventually HD immediately after the procedure for adequate ultrafiltration), the minimum necessary dose, prophylactic treatment with acetilcisteine [69,70], preferring hypo–osmolar non–ionic contrast substances [69]. In PD patients, the administration of aminoglycosides is accompanied in some of the studies by an acceleration of RRF decline [71], while in other studies [72,73] it had no influence, which is explainable through the intermittent administration of intraperitoneal treatments. A recent study [73] compared the influence on RRF of intraperitoneal regimen for peritonitis with ceftazidime versus netilmicine (6 weeks, administrated once daily) and didn't find any difference.

The administration of diuretics in chronic dialysed patients is accompanied by an increase of diuresis and decrease of hidrosaline retention, but doesn't influence RRF [74,75,76].

ACEs inhibitors or/and angiotensin receptors blockers may have the same renoprotective effects as in predialysis [77,78]. Regardless of the drug regimen, controlling systemic hypertension is accompanied by longer preservation of RRF. In the same time, performing excessive ultrafiltration compromises in time RRF [79,80]. Therefore, excessive ultrafiltration, intradialytic hypotension and semnificative interdialytic weight gain should be avoided.

## References

[1] Misra M, Vonesh E, Churchill DN, Moore HL, Van Stone JC, Nolph KD (2000). Preservation of glomerular filtration rate od dialysis when adjusted for patient dropout. Kideny Int.

[2] Donadio C, Caprio F, Grassi G, Barsotti G (2005). Evaluation of residual renal function in end-stage renal diseases patients (CKD stage 5). J Am Soc Nephrol (Nov).

[3] Ixkes MC, Koompan MG, Van Acker BA, Weber JA, Arisz L (1997). Cimetidine improves GFR-estimation by Cockcroft and Gault formula. Clin. Nephrol.

[4] Kemperman C, Caprio FAW, Krediet RT, Arisz L (2002). Formula-derived prediction of the glomerular filtration rate from plama creatinine concentration. Nephron.

[5] Levey AS, Bosch JP, Lewis JG, Green T, Rogers N, Roth D (1999). Formula for the modification of diet in renal disease study group. A more accurate method to estimate glomerular 
filtration rate from serum creatinine: a new prediction equation. Ann Inter Med.

[6] Simonsen O, Grubb A, Thyssel H (1985). blood serum concentration of cystatin-C (gamma-trace) as a measure of the glomerular filtration rate. Scand J Clin Lab Invest.

[7] Van Olden  RW, Krediet RT, Struijk DG, Arisz L (1996). Measurement of residual renal function in patients treated with CAPD.. J Am Soc Nephrol.

[8] Swan SK, Halstenson CE, Kasiske BL, Collins AJ (1996). Determination of residual renal function with iohexol clearance in hemodialysis patients.. Kideny Int.

[9] Maiorca R, Brunori G, Zubani R (1995). Predictive value of dialysis adequacy and nutritional 
indices for mortality and morbidity in CAPD and HD patients. A longitudinal
study.. Kideny Int.

[10] Buxo JA, Lowrie EG, Lew NL (1999). Associates of mortality among peritoneal dialysis patients with special reference to peritoneal transport rates and solute
clearance. Am J Kidney Dis.

[11] Rocco M, Soucie JM, Pastan S, McClellani WM (2000). Peritoneal dialysis adequacy and risk of death. Kideny Int.

[12] rocco MV, Frankenfield DL, Prowant B, Frederick P, Flanigan MJ (2002). centers for Medicare and Medicaid Services Peritoneal Dialysis Core Indicators Study Group. Risk factors for early 
mortality in U.S. peritoneal dialysis patients: impact of residual renal function. Perit Dial Int..

[13] (1996). Adequacy of dialysis and nutrition in continuous peritoneal dialysis: association with clinical outcomes. Canada–USA 
(CANUSA) Peritoneal Dialysis Study Group. Kideny Int.

[14] Bargman JM, Thorpe KE, Churchill DN (2001). Relative contribution of residual renal function and peritoneal clearance to adequacy of dialysis: a reanalysis of the 
CANUSA study. Am J Kidney Dis.

[15] shemin D, Bostom AG, Laliberty P, Dworkin LD (2001). Residual renal function and mortality risk in hemodialysis patients.. Am J Kidney Dis.

[16] Termorshuizen F, Dekker FW, van Manen  JG (2004). Relative contribution of residual renal function and different measures of adequacy to survival in hemodialysis patients: 
an analysis of the Netherlands Cooperative Study on the Adequacy of Dialysis (NECOSAD)-2.. Kideny Int.

[17] Piniagua R, Amato D, Vonesh E (2002). Effects of increased peritoneal clearances on mortality rates in peritoneal dialysis: ADEMEX, a prospective, randomized, 
controlled trial.. J Am Soc Nephrol.

[18] Wang Ay, Woo J, Wang M (2005). Important differentiation of factors that predict outcome in peritoneal dialysis patients with different degrees of 
residual renal function. Nephrol Dial Transplant.

[19] Konings CJ, Kooman JP, Schonck M (2003). Fluid status in CAPD patients is related to peritoneal transport and residual renal function: evidence from a longitudinal
 study.. Nephrol Dial Transplant.

[20] Ates K, Nergizoglu G, Keven K (2001). Effect of fluid and sodium removal on mortality in peritoneal dialysis patients.. Kideny Int.

[21] Wang AY, Sanderson J, Sea MM (2003). Important factors other than dialysis adequacy associated with inadequate dietary protein and energy intakes in patients 
receiving maintenance peritoneal dialysis.. Am J Clin Nutr.

[22] Wang AY, Wang M, Woo J (2002). A novel association between residual renal function and left ventricular hypertrophy in peritoneal dialysis patients.. Kideny Int.

[23] Menon MK, Naimark DM, Bargman JM (2001). Long-term blood pressure control in a cohort of peritoneal dialysis patients and its association with residual renal 
function. Nephrol Dial Transplant.

[24] Levin A, Thompson CR, Ethier J (1999). Left ventricular mass index increase in early renal disease: impact of decline in hemoglobin.. Am J Kidney Dis.

[25] Leenen FH, Smith DL, Khnanna R, Orepoulos DG (1985). Changes in left ventricular hypertrophy and function in hypertensive patients started on continuous ambulatory peritoneal 
dialysis. Am Heart J.

[26] Rigatto C, Foley RN, Kent GM (2000). LLong-term changes in left ventricular hypertrophy after renal transplantation. Transplantation.

[27] Fagugli RM, Pasini P, Quintaliani G (2003). Association between extracellular water, left ventricular mass and hypertension in haemodialysis patients.. Nephrol Dial Transplant.

[28] Gunal Al, Kirciman E, Guler M, Yavuzkir M, Celiker H (2004). Should the preservation of residual renal function cost volume overload and its consequence left ventricular hypertrophy 
in new hemodialysis patients?. Ren Fail.

[29] Winchester JF, Rottelar C, Goggins M (1993). Calcium and phosphate balance in dialysis patients.. Kidney Int Suppl.

[30] Wang AY, Woo J, Sea MM (2004). Hyperphosphatemia in Chinese peritoneal dialysis patients with and without residual kidney function: what are the 
implications?. Am J Kidney Dis.

[31] Wang AY, Woo J, Lam CW (2005). Associations of serum fetuin-A with malnutrition, inflammation, atherosclerosis and valvular calcification syndrome and 
outcome in peritoneal dialysis patients. Nephrol Dial Transplant.

[32] Arici M, wALLS j (2001). End-stage renal disease, atherosclerosis, and 
cardiovascular mortality: is C-reactive protein the missing link?. Kidney Int.

[33] Wang AY, Woo J, Lam CW (2003). Is a single time point C-reactive protein predictive of outcome in peritoneal dialysis patients?. J Am Soc Nephrol.

[34] Zimmermann J, Herrlinger S, Pruy A (1999). Inflammation enhances cardiovascular risk and mortality in hemodialysis patients.. Kidney Int.

[35] Panich V, Maggiore U, Bargman JM, Taccola D (2004). Interleukin-6 is a stronger predictor of total and cardiovascular mortality than C-reactive protein in haemodialysis 
patients.. Nephrol Dial Transplant.

[36] Rao M, Guo D, Perianayagam MC (2005). Plasma interleukin-6 predicts cardiovascular mortality in hemodialysis patients.. Am J Kidney Dis.

[37] Wang AY, Wang M, Woo J (2004). Inflammation, residual kidney function, and cardiac hypertrophy are interrelated and combine adversely to enhance 
mortality and cardiovascular death risk of peritoneal dialysis patients.. J Am Soc Nephrol.

[38] Wang AY, Lam CW, Wang M (2005). Circulating soluble vascular cell adhesion molecule 1: relationships with residual renal function, cardiac hypertrophy, 
and outcome of peritoneal dialysis patients.. Am J Kidney Dis.

[39] Chung SH, Heimburger O, Stenvinkel P (2001). Association between inflammation and changes in residual renal function and peritoneal transport rate during the first 
year of dialysis.. Nephrol Dial Transplant.

[40] Pecoits-Filho  R, Heimburger O, Barany P (2003). Associations between circulating inflammatory markers and residual renal function in CRF patients.. Am J Kidney Dis.

[41] Shlipak MG, Fried LF, Crump C (2003). . Elevations of inflammatory and procoagulant biomarkers in elderly persons with renal insufficiency.. Circulation.

[42] Witko-Sarsat  V, Friedlander M, Nguyen KT (1998). Advanced oxidation protein products as novel mediators of inflammation and monocyte activation in chronic renal failure.. J Immunol.

[43] Bemelmans MH, Gouma DJ, Buurman WA (1993). Influence of nephrectomy on tumor necrosis factor clearance in a murine model.. J Immunol.

[44] Shin SK, Noh H, Kang SW (1999). Risk factors influencing the decline of residual renal function in continuous ambulatory peritoneal dialysis patients.. Perit Dial Int.

[45] Wang AY, Sea MM, Ip R (2001). Independent effects of residual renal function and dialysis adequacy on actual dietary protein, calorie, and other 
nutrient intake in patients on continuous ambulatory peritoneal dialysis.. J Am Soc Nephrol.

[46] Wang AY, Sea MM, Ho ZS (2005). Evaluation of handgrip strength as a nutritional marker and prognostic indicator in peritoneal dialysis patients.. Am J Clin Nutr.

[47] Suda T, Hiroshige K, Ohta T (2000). The contribution of residual renal function to overall nutritional status in chronic haemodialysis patients.. Nephrol Dial Transplant.

[48] Wang AY, Sea MM, Ip R (2002). Independent effects of residual renal function and dialysis adequacy on dietary micronutrient intakes in patients 
receiving continuous ambulatory peritoneal dialysis.. Am J Clin Nutr.

[49] Wang AY, Sea MM, Tang N (2004). Resting energy expenditure and subsequent mortality risk in peritoneal dialysis patients.. J Am Soc Nephrol.

[50] Maeda K, Shinzato T, Ota T (1990). Beta-2-microglobulin generation rate and clearance rate in maintenance hemodialysis patients.. Nephron.

[51] Amici G, Virga G, Da RG (1993). Serum beta-2-microglobulin level and residual renal function in peritoneal dialysis.. Nephron.

[52] Brown PH, Kalra PA, Turney JH, Cooper EH (1988). Serum low-molecular-weight proteins in haemodialysis patients: effect of residual renal function.. Nephrol Dial Transplantt.

[53] Bammens B, Evenepoel P, Verbeke K, Vanrenterghem Y (2005). Time profiles of peritoneal and renal clearances of different uremic solutes in incident peritoneal dialysis patients.. Am J Kidney Dis.

[54] Bammens B, Evenepoel P, Verbeke K, Vanrenterghem Y (2003). Removal of middle molecules and protein-bound solutes by peritoneal dialysis and relation with uremic symptoms. Kidney Int.

[55] Termorshuizen F, Korevaar JC, Dekker FW (2003). The relative importance of residual renal function compared with peritoneal clearance for patient survival and quality of 
life: an analysis of the Netherlands Cooperative Study on the Adequacy of Dialysis (NECOSAD)-2. Am J Kidney Dis.

[56] Rottembourg J, Issad B, Gallego JL (1983). Evolution of residual renal function in patients undergoing maintenance haemodialysis or continuous ambulatory peritoneal 
dialysis. Proc Eur Dial Transplant Assoc.

[57] Misra M, Vonesh E, Van Stone JC (2001). Effect of cause and time of dropout on the residual GFR: a comparative analysis of the decline of GFR on dialysis.. Kidney Int.

[58] Lysaght MJ, Vonesh EF, Gotch F (1991). The influence of dialysis treatment modality on the decline of remaining renal function.. ASAIO Trans.

[59] Moist LM, Port FK, Orzol SM (2000). Predictors of loss of residual renal function among new dialysis patients.. J Am Soc Nephrol.

[60] Van BW, Vanholder RC, Veys N (2000). An evaluation of an integrative care approach for end-stage renal disease patients.. J Am Soc Nephrol.

[61] McKane W, Chandna SM, Tattesall JE (2002). Identical decline of residual renal function in high-flux biocompatible hemodialysis and CAPD.. Kidney Int.

[62] Schiffl H, Lang SM, Fischer R (2002). Ultrapure dialysis fluid slows loss of residual renal function in new dialysis patients.. Dial Transplant.

[63] Stannat S, Bahlmann J, Kiessling D (1985). Complement activation during hemodialysis. Comparison of polysulfone and cuprophan membranes.. Contrib Nephrol.

[64] Holley JL, Aslam N, Bernardini J (2001). The influence of demographic factors and modality on loss of residual renal function in incident peritoneal dialysis 
patients.. Perit Dial Int.

[65] Hufnagel G, Michel C, Queffeulou G (1999). The influence of automated peritoneal dialysis on the decrease in residual renal function.. Nephrol Dial Transplant.

[66] Van BW, Vanholder R, Veys N, Lameire N (2002). The role of APD in the improvement of outcomes in an ESRD program.. Semin Dial.

[67] Rodriguez-Carmona A, Perez-Fontan M, Garca-Naveiro R (2004). Compared time profiles of ultrafiltration, sodium removal, and renal function in incident CAPD and automated peritoneal 
dialysis patients.. Am J Kidney Dis.

[68] Aspelin P, Aubry P, Fransson SG (2003). Nephrotoxic effects in high-risk patients undergoing angiography. N Engl J Med.

[69] Tepel M, van der GM, Schwarzfeld C (2000). Prevention of radiographic-contrast-agent-induced reductions in renal function by acetylcysteine. N Engl J Med.

[70] Birck R, Krzossok S, Markowetz F (2003). Acetylcysteine for prevention of contrast nephropathy. meta-analysis. Lancet.

[71] Shemin D, Maaz D, St PD (1999). Effect of aminoglycoside use on residual renal function in peritoneal dialysis patients.. Am J Kidney Dis.

[72] Baker RJ, Senior H, Clemenger M, Brown EA (2003). Empirical aminoglycosides for peritonitis do not affect residual renal function.. Am J Kidney Dis.

[73] Lui SL, Cheng SW, Ng F (2005). Cefazolin plus netilmicin versus cefazolin plus ceftazidime for treating CAPD peritonitis: effect on residual renal 
function.. Kidney Int.

[74] Medcalf JF, Harris KP, Walls J (2001). ARole of diuretics in the preservation of residual renal 
function in patients on continuous ambulatory peritoneal dialysis.. Kidney Int.

[75] Van Olden RW, Guchelaar HJ, Struijk DG (2003). Acute effects of high-dose furosemide on residual renal function in CAPD patients.. Perit Dial Int.

[76] Van Olden RW, Van Meyel JJ, Gerlag PG (1995). Sensitivity of residual nephrons to high dose of furosemide described by diuretic efficiency.. Eur J Clin Pharmacol.

[77] Li PK, Chow KM, Wong TY (2003). Effects of an angiotensin-converting enzyme inhibitor on residual renal function in patients receiving peritoneal 
dialysis. A randomized, controlled study. Ann Intern Med.

[78] Suzuki H, Kanno Y, Sugahara S (2004). Effects of an angiotensin II receptor blocker, valsartan, 
on residual renal function in patients on CAPD.. Am J Kidney Dis.

[79] Gunal AI, Duman S, Ozkahaya M (2001). Strict volume control normalizes hypertension in peritoneal
dialysis patients.. Am J Kidney Dis.

[80] Lameire N (2005 nov). Preservation of residual renal function in hemodialysed 
patients.. ASN Renal Week, Clinical Nephrology Conferences.

